# Modeling Somatic Mutations Associated With Neurodevelopmental Disorders in Human Brain Organoids

**DOI:** 10.3389/fnmol.2021.787243

**Published:** 2022-01-04

**Authors:** Bipan K. Deb, Helen S. Bateup

**Affiliations:** ^1^Department of Molecular and Cell Biology, University of California, Berkeley, Berkeley, CA, United States; ^2^Helen Wills Neuroscience Institute, University of California, Berkeley, Berkeley, CA, United States; ^3^Chan Zuckerberg Biohub, San Francisco, CA, United States

**Keywords:** human brain organoids, neurodevelopmental disorders, mTOR, somatic mutations, cortical development, malformations of cortical development

## Abstract

Neurodevelopmental disorders (NDDs) are a collection of diseases with early life onset that often present with developmental delay, cognitive deficits, and behavioral conditions. In some cases, severe outcomes such as brain malformations and intractable epilepsy can occur. The mutations underlying NDDs may be inherited or *de novo*, can be gain- or loss-of-function, and can affect one or more genes. Recent evidence indicates that brain somatic mutations contribute to several NDDs, in particular malformations of cortical development. While advances in sequencing technologies have enabled the detection of these somatic mutations, the mechanisms by which they alter brain development and function are not well understood due to limited model systems that recapitulate these events. Human brain organoids have emerged as powerful models to study the early developmental events of the human brain. Brain organoids capture the developmental progression of the human brain and contain human-enriched progenitor cell types. Advances in human stem cell and genome engineering provide an opportunity to model NDD-associated somatic mutations in brain organoids. These organoids can be tracked throughout development to understand the impact of somatic mutations on early human brain development and function. In this review, we discuss recent evidence that somatic mutations occur in the developing human brain, that they can lead to NDDs, and discuss how they could be modeled using human brain organoids.

## Introduction

Neurodevelopmental disorders (NDDs) impact brain development and function resulting in a range of neurological and psychiatric manifestations. These can include epilepsy, intellectual disability, autism spectrum disorder (ASD), and other behavioral, cognitive, and affective disorders (Niemi et al., 2018; Parenti et al., 2020). Individuals with NDDs present with an array of clinical symptoms early in life suggesting that multiple neural circuits are impacted during both pre- and postnatal brain development.

Extensive genetic mapping over the years has identified mutations in hundreds of genes that are associated with NDDs (Gilissen et al., 2014; Tarlungeanu and Novarino, 2018; Satterstrom et al., 2020; Wang et al., 2020). In particular, genes associated with syndromic NDDs that have multiple neuropsychiatric and medical presentations often encode proteins that control essential cellular functions including the regulation of gene expression (e.g., *CDH8, MECP2, SETD5*), RNA processing (e.g., *FMR1*), protein synthesis (e.g., *TSC1/2, PTEN*), and protein degradation (e.g., *UBE3A*). Mutations in genes encoding proteins important for neuronal excitability and synaptic transmission are also frequently found in NDDs including ASD, epileptic encephalopathy, and intellectual disability (e.g., *SYNGAP1, SHANK3, GRIN2B, SCN1A, SCN2A*; Parenti et al., 2020). How these mutations alter nervous system development, synaptic connectivity, and neural circuit activity is an ongoing area of research. Mouse models of NDDs harboring mutations in these genes have revealed an array of developmental, cellular, synaptic, and behavioral phenotypes that have provided insight into the basic functions of these genes and how changes in their expression may lead to NDDs (Verma et al., 2019; Bozzi and Fagiolini, 2020; Karalis and Bateup, 2021). In particular, studying these genes in animal models enables an understanding of the impact of mutations across multiple levels from molecules to circuits to behavior. However, it remains an open question whether mechanisms uncovered in animal models are translatable to humans. Thus, our knowledge of how mutations in these genes affect human neural development, synaptic connectivity, and circuit dynamics is still limited.

Recent advances in human cell reprogramming and genome engineering have facilitated the development of models that enable the study of disease mutations in a human genetic and cellular context. Such systems provide a valuable complement to animal models to advance our understanding of disease mechanisms.

With this approach, human embryonic (hESC) or induced pluripotent (hiPSC) stem cells (collectively hPSCs) can be differentiated into various types of neurons and glia. *In vitro* human neuronal models have traditionally been 2D monolayer cultures; however, it is technically challenging to maintain these cultures for longer than a few weeks or months. Maturation of neural circuits in the developing human brain occurs over 9 months of gestation and for several years postnatally (Stiles and Jernigan, 2010). Thus, *in vitro* models that can be maintained long-term are required to capture this time-dependent maturation, which is highly relevant to the study of NDDs. Over the last several years, this has been achieved by harnessing the ability of hPSCs to form 3D aggregates when maintained in suspension (Paşca, 2019). The formation of 3D structures allows neuronal and glial differentiation and development over prolonged periods of time (up to 600 days and beyond). This enables the generation of greater cellular diversity and important developmental transitions to be captured *in vitro* (Gordon et al., 2021). Therefore, these 3D “human brain organoids” (hBOs) provide an opportunity to model and understand basic principles of early human brain development in both normal and pathological states.

In this mini-review, we discuss the utility of hBO models for studying brain somatic mutations during development, which can directly cause certain NDDs and may be an important contributor to many other neuropsychiatric disorders (D’gama and Walsh, 2018; Jourdon et al., 2020). We describe emerging evidence that brain somatic mutations contribute to NDDs and discuss how hBOs provide an opportunity to model and understand the consequences of somatic mutations during the early stages of human brain development.

## Brain Somatic Mutations and Their Contribution to Neurodevelopmental Disorders

Somatic mutations arise post-fertilization. During development they can occur in a progenitor cell and be passed on to the daughter cells forming a cluster of cells with the mutation surrounded by cells without the mutation, which are derived from different progenitors. Somatic mutations can also occur in post-mitotic cells and it is estimated that human neurons acquire ~23 single-nucleotide variants (SNVs) per year over the lifespan of an individual (D’gama and Walsh, 2018). Somatic mutations affect only a fraction of cells in an organism, in contrast to inherited mutations, which are present in every cell of the offspring. The number and type of cells affected depend on the origin and developmental timing of the mutation (D’gama and Walsh, 2018; Dou et al., 2018). A mutation occurring early in development can affect many cells across multiple tissues, while a mutation occurring later may only affect a small number of cells within a specific lineage. Somatic mutations can arise due to errors during DNA replication (Rogozin et al., 2001); therefore actively dividing cells are more vulnerable to acquiring somatic mutations. Somatic mutations have been traditionally studied in cancer where they can act as oncogenic drivers (Greenman et al., 2007). Because of the limited number of cells that are affected, identifying somatic mutations is challenging. Low alternate allelic frequency (AAF) means that high DNA sequencing depth is required to identify somatic mutations in bulk tissue. Notably, AAFs as low as 1%, indicating that 1% of cells in a tissue sample carry the mutation, can be sufficient to cause disease (D’gama et al., 2017).

In the human cortex, an extended period of neurogenesis that spans ~6 months gives rise to the diversity of neuron types that make up the cortical layers (Silbereis et al., 2016). Later in development, cortical progenitors begin producing astrocytes and other glial cell types. This protracted period of corticogenesis, which requires huge numbers of cell divisions to produce the ~86 billion neurons of the human cortex (Azevedo et al., 2009), likely increases the probability of acquiring somatic mutations. Indeed, somatic mosaicism has been detected in human brain tissue and it is estimated that a given neuron in the adult human brain may have 800–2,000 somatic single nucleotide variants (Lodato et al., 2015). While some of these mutations contribute to brain diseases, somatic mutations have also been hypothesized to contribute to neuronal diversity and inter-individual heterogeneity of the human brain (Jourdon et al., 2020).

**Figure 1:**
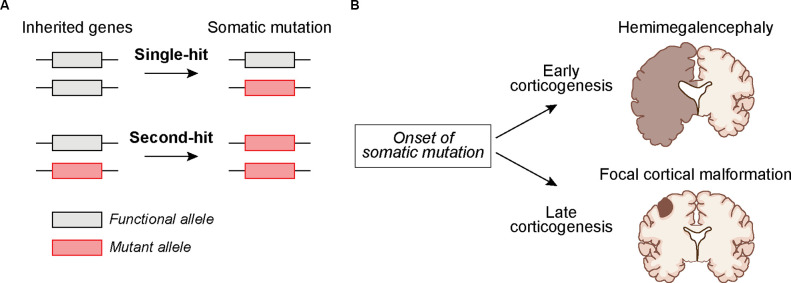
Potential mechanisms by whichsomatic mutations during development can lead to cortical malformations. **(A)** The mutation may occur in cortical progenitors as a “single-hit” in individuals with no inherited mutation or as a “second-hit” in individuals with an inherited mutation. **(B)** The timing of somatic mutations can determine the outcome on cortical development. A somatic mutation acquired early during corticogenesis can lead to malformations that affect an entire cortical hemisphere, as in Hemimegalencephaly. Somatic mutations acquired later in development may lead to more localized cortical malformations as seen in Focal Cortical Dysplasia and TSC-associated cortical tubers.

Identifying brain somatic mutations is especially challenging given that human brain tissue is inaccessible and usually only available post-mortem. Exceptions to this are cases in which brain tissue is surgically removed to control intractable epilepsy (Sim et al., 2019; Grayson et al., 2020). This is typically done in cases in which seizures can be mapped to focal lesions. Through the analysis of this surgically resected tissue, somatic mutations have been identified in genes that are part of the greater mTOR signaling network (Crino, 2020). mTOR is part of two protein complexes, mTORC1 and mTORC2, which serve as central coordinators of intra- and extracellular signals that control cell growth, morphology, and metabolism (Saxton and Sabatini, 2017; Liu and Sabatini, 2020). Mutations associated with cortical malformations and seizures often cause hyperactivation of mTORC1 signaling either *via* gain-of-function mutations in positive regulators of mTORC1 (e.g., *RHEB, AKT3, PIK3CA*, and *MTOR* itself), or loss-of-function mutations in negative regulators (e.g., *PTEN*, *TSC1*, *TSC2, STRADA, DEPDC5, NPRL3, NPRL2*) (Puffenberger et al., 2007; Lee et al., 2012; Lim et al., 2015, 2017b; Ribierre et al., 2018; Kim et al., 2019; Zhao et al., 2019; Ye et al., 2019; Koboldt et al., 2021). Mutations in mTOR pathway genes can also occur in the germline giving rise to systemic NDDs that affect multiple organ systems, such as in Tuberous Sclerosis Complex (TSC). In this case, the germline mutation is heterozygous and a somatic “second-hit” mutation that disrupts the functional allele can occur, leading to focal brain lesions including cortical tubers and benign growths called subependymal nodules (SENs) and subependymal giant cell astrocytomas (SEGAs; Crino et al., 2010; Figure 1). Second-hit mutations were originally described in cancer (Knudson, 1971) and have been found in TSC-associated tumors, called hamartomas, in the skin, kidney, and other tissues (Henske et al., 1996; Sepp et al., 1996; Au et al., 1999). Second-hit mutations have also been identified in SEGAs and in some but not all resected cortical tubers (Chan et al., 2004; Crino et al., 2010; Qin et al., 2010; Martin et al., 2017). Notably, mutations in *TSC1* and *TSC2* can also occur somatically in the absence of a germline mutation, leading to focal brain lesions in the absence of systemic manifestations (D’gama et al., 2015, 2017; Lim et al., 2017b; Figure 1).

In addition to “mTORopathies”, which are associated with cortical malformations and epilepsy (Karalis and Bateup, 2021), brain somatic mutations have been identified in tissue from individuals with a variety of disorders including Rett syndrome, neurofibromatosis type 1, neuronal migration disorders, epileptic encephalopathies, ASD, intellectual disability, schizophrenia, and neurodegenerative diseases (Muotri et al., 2010; Garcia-Linares et al., 2011; D’gama and Walsh, 2018; Park et al., 2019; Kim et al., 2021; Rodin et al., 2021). In particular, several recent studies have shown that brain somatic mutations account for approximately 3–5% of ASD cases from the Simons Simplex Collection (Freed and Pevsner, 2016; Dou et al., 2017; Krupp et al., 2017; Lim et al., 2017a). The somatic mutations identified thus far may only be the tip of the iceberg with more to be discovered with improved sequencing technology, bioinformatics, and access to human brain tissue (Jourdon et al., 2020; Wang et al., 2021).

## Brain Organoids as Models of Human Embryonic Brain Development

hBOs have emerged as powerful models to study early human brain development. Undirected neural differentiation of hPSCs, in the absence of specific patterning cues beyond initial neural induction, gives rise to hBOs containing cells of multiple neural lineages that can include forebrain, ganglionic eminences, midbrain, retina, and others, in a manner that is stochastic and can differ from organoid to organoid (Lancaster et al., 2013; Quadrato et al., 2017). Directed differentiation protocols have been developed to generate hBOs that more reproducibly give rise to cell lineages of a particular brain region like the cortex (Kadoshima et al., 2013; Pasca et al., 2015; Qian et al., 2018; Pollen et al., 2019; Velasco et al., 2019), midbrain (Jo et al., 2016), striatum (Miura et al., 2020), or cerebellum (Ballabio et al., 2020). Human cortical organoids have been used widely and recapitulate some, but not all, of the cellular diversity and intrinsic developmental timing of the early stages of human cortical development (Di Lullo and Kriegstein, 2017). Notably, cortical organoids contain outer radial glia cells (oRGs), which are a highly proliferative progenitor cell type enriched in the human fetal cortex (Pollen et al., 2015). Cortical organoids are therefore a good model for assessing the impact of NDD-associated mutations on the biology of oRGs, which is not possible in other model systems, including mice, which lack significant numbers of this progenitor cell type. Early in the differentiation process, cortical organoids also capture some of the structural features of cortical development including the generation of ventricular and subventricular zone-like cellular assemblies, which give rise to a surrounding rudimentary cortical plate that can include areas containing upper and lower layer cortical neurons (Qian et al., 2020). Importantly, these organoids recapitulate the temporal segregation of neurogenesis and astrogenesis that occurs in the developing human cortex, with neurons born first and astrocytes emerging around 3–4 months post-differentiation (Pasca et al., 2015; Sloan et al., 2017). Interestingly, astrocytes continue to develop in brain organoids over time and spontaneously transition to a more mature “postnatal”-like state after about 7–9 months in culture (Sloan et al., 2017).

While hBOs offer several advantages to modeling the developing human brain, there are important challenges associated with hBOs that necessitate optimization of differentiation protocols and establishing standards for quality control and reproducibility. For example, hBOs generated from different hESC or hiPSC lines may differ in their potential to differentiate into particular cell lineages or brain regions. Since hPSCs need to be differentiated in batches, there can be batch-to-batch variability in differentiation or cell health even when starting from the same hPSC line. Continued culturing of hPSCs may lead to the acquisition of genetic or epigenetic changes that could affect differentiation capacity or cellular phenotypes. Since hBOs do not have a blood supply, there may be limited access to oxygen and nutrients, especially for cells in the center of the organoid when it reaches a certain size. If this activates cellular stress responses, it may interfere with proper differentiation (Bhaduri et al., 2020). While technological advancements are continuously arising to address these challenges, for example slicing hBOs to maintain access to oxygen (Qian et al., 2020) or implanting hBOs into a rodent host brain (Mansour et al., 2018), careful consideration needs to be given to reproducibility and quality control when working with hPSCs and brain organoids.

## Gene-Edited and Patient-Derived hPSCs for Modeling Neurodevelopmental Disorders

NDDs can be modeled in hBOs using hPSCs that have been gene-edited to disrupt the expression of an NDD-associated gene or introduce a disease-associated mutation. This is relevant in the case of NDDs for which causal or high-confidence risk genes have been identified. Alternatively, hiPSCs reprogrammed from patient cells can be used to generate hBOs. This is beneficial for NDDs in which multiple or unknown genetic variants are involved. Recapitulating this complex patient-specific genetic landscape by gene-editing would be difficult and hence hiPSCs provide a useful resource in this regard. hBOs generated from patient-derived hiPSCs have been used to understand the cellular mechanisms underlying the cortical malformations observed in Miller-Dieker and Pretzel syndromes (Bershteyn et al., 2017; Iefremova et al., 2017; Dang et al., 2021). iPSC-derived hBOs have also been used to identify changes in neuronal composition and molecular alterations associated with Rett syndrome (Gomes et al., 2020; Samarasinghe et al., 2021), idiopathic ASD (Mariani et al., 2015; Chiola et al., 2021), microcephaly (Lancaster et al., 2013), schizophrenia (Stachowiak et al., 2017; Khan et al., 2020), and other disorders. However, patient-to-patient genetic variability can be a challenge for directly linking cellular phenotypic outcomes to a specific genetic cause. In addition, defining an appropriate control group is an issue due to genetic heterogeneity within the human population. One way to address this is to correct the NDD-associated patient mutation using gene editing to generate an isogenic control line (Soldner and Jaenisch, 2018).

A limitation to the use of hiPSCs is that somatic brain mutations, which may drive or modulate NDD phenotypes in some cases, are not normally present in patient-derived blood cells or fibroblasts that are used to generate hiPSCs. Furthermore, the opposite complication can arise whereby somatic mutations acquired in patient skin fibroblasts that are used for reprogramming could lead to phenotypes in hBOs that are not related to the brain manifestations of the disease (Abyzov et al., 2012). Therefore, assessing how somatic mutations impact human brain development requires additional approaches and considerations.

## Modeling Somatic Mutations in Brain Organoids

During development, a somatic mutation can occur in a single progenitor, which will then pass it on to its progeny, assuming that it is not deleterious for cell survival. While the neurons and glia differentiated from progenitors with an acquired mutation may directly cause a brain malformation, as in focal cortical dysplasia, it is possible that the mutant cells could also alter the development and function of the surrounding “normal” cells *via* non-cell autonomous interactions. Therefore, systems that capture this genetic mosaicism are needed to model somatic mutations in hBOs and study their autonomous and non-cell autonomous impact on brain development.

There are several ways in which the consequences of a disease-associated mutation have been studied in a genetically heterogeneous context in hBOs. For each of these approaches, it is necessary to distinguish cells of different genotypes and fluorescent labels can be useful for this. Due to the mosaic nature of these organoids, approaches that assess the differentiation, morphology, connectivity, physiology, or gene expression profile of individual cells are advantageous as bulk analyses of the entire organoid would be difficult to interpret.

**Figure 2:**
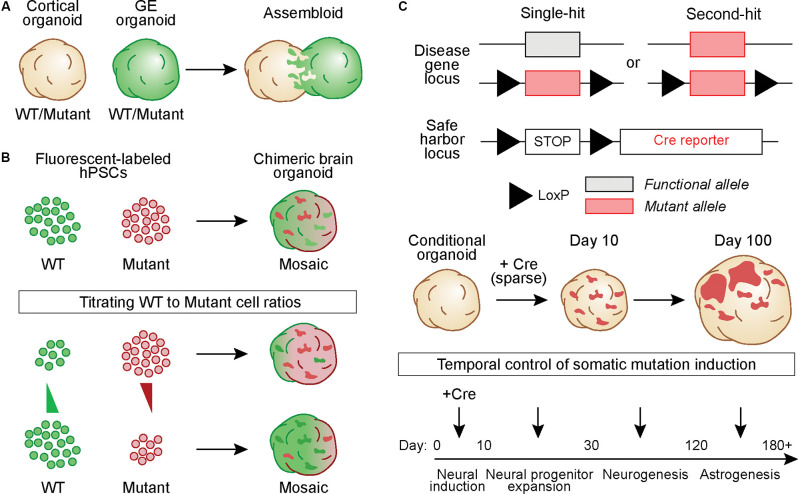
Generating hBOs that capture the development and pathological outcomes of brain somatic mutations. **(A)** Chimeric assembloids can be generated by fusing cortical and ganglionic eminence (GE) organoids derived from eitherwild-type (WT) or mutant hPSCs, in different combinations. In this example, cells in the GE organoid express a green fluorescent protein. **(B)** WT and mutant hPSCs that express different fluorescent proteins can be mixed to generate chimeric hBOs. The proportion of cells of each genotype can be adjusted to create mosaic hBOs with different ratios of WT to mutant cells. Such an approach can be used to model different alternate allelic frequencies. **(C)** hPSCs can be gene-edited to generate Cre-inducible conditional mutations. Cells can harbor one copy of the conditional allele, in which Cre induces a heterozygous mutation (“Single-hit”), or have one constitutive mutant allele and one conditional allele, thus causing complete loss of function when Cre is expressed (“Second-hit”). These hPSCs can be further gene-edited to include a LoxP-STOP-LoxP cassette followed by a fluorescent reporter in a safe harbor locus. This allows labeling and tracking of all conditional mutant cells and their progeny. hBOs generated from these conditional lines can betransduced with Cre at varying time points during development to vary the timing of the simulated somatic mutation.

In one approach, hBOs derived from a fusion of cortex (Cx) and ganglionic eminence (GE) organoids called “assembloids” were generated from healthy and Timothy Syndrome patient-derived iPSCs to study defects in interneuron migration (Birey et al., 2017). Similar fusion organoids have been generated from Rett syndrome patient-derived iPSCs in which either the Cx or GE organoid carried the *MECP2* mutation. This study used extracellular recordings and calcium imaging to show that patient-derived mutant interneurons in the GE organoid could modulate circuit activity in the fused cortical organoid, which contained cells from a healthy donor (Samarasinghe et al., 2021; Figure 2A). While these studies were not designed to model somatic mutations *per se*, they show how a mutation in a sub-population of developing neurons can non-cell autonomously alter the development and activity of adjacent, normally developing cells.

In another approach, a cell mixing strategy can be used to generate chimeric hBOs containing wild-type (WT) and mutant progenitor cells expressing different fluorescent reporters (Figure 2B). With this strategy, it is possible to modulate the ratio of WT to mutant cells and study the effects of varying cellular compositions on a range of phenotypic outcomes such as differentiation capacity, gene expression, morphology, and electrophysiology. Such a strategy would be relevant to NDDs such as lissencephaly and double cortex syndrome in which the percentage of cells harboring the mutant gene results in differing outcomes, ranging from more mild subcortical band heterotopia with low AAF (i.e., ~10%) to severe complete lissencephaly with a germline mutation (100% of cells expressing the mutation; Jamuar et al., 2014; D’gama and Walsh, 2018).

Another strategy for modeling a somatic mutation is to use Cre recombinase to induce a conditional mutation in developing hBOs (Figure 2C). This can be done using sparse viral delivery of Cre to hBOs derived from cells that are heterozygous or homozygous for a conditional mutation. Cells that are transduced by Cre will harbor the mutated allele and be surrounded by WT cells, modeling a genetically-mosaic developing brain. An extension of this approach can be used to model a somatic “second-hit” mutation. In this case, the hBOs are generated from hPSCs carrying a constitutive NDD-associated mutation in one allele and a Cre-inducible mutation in the second allele. In the presence of Cre, the functional allele is disrupted thereby generating a “second-hit”. Such an approach has been used in mouse models of TSC (Feliciano et al., 2011), where *in utero* electroporation was used to deliver Cre to a small population of *Tsc1^c/-^* cortical progenitors during embryonic development, resulting in the formation of a focal region of dysplastic cells. More recently, a similar approach was used in *TSC2^c/-^* hBOs with lentivirus-mediated delivery of Cre to conditionally delete *TSC2* from a small population (~1–5%) of neural progenitors early in development (Blair et al., 2018). This resulted in focal regions within the organoid that when analyzed by immunohistochemistry contained dysmorphic and enlarged cells with altered differentiation capacity, resembling cortical tuber-like lesions (Blair et al., 2018; Figure 2C).

Delivering Cre to hBOs with a virus allows temporal control over the conditional mutation by modulating the timing of Cre expression, thereby generating mutant progenitors or post-mitotic cells at different stages of development (Figure 2C). Differences in the timing of the somatic mutation may lead to differential outcomes on the cellular composition and severity of the lesions formed. This is particularly relevant in the context of human cortical neurogenesis, which spans ~6 months and proceeds through temporally segregated periods of development during which progenitors transition to produce a variety of cell fates (Lui et al., 2011). Therefore, introducing the somatic mutation at different time-points of hBO development may reveal mechanisms that control the cellular composition of focal lesions and their impact on neural circuit development and function.

## Conclusions

Understanding the cellular and molecular mechanisms of human brain development has been greatly challenged by limited access to developing human brain tissue. While model organisms are instrumental for studying the fundamentals of neural development, circuit function, and behavioral control, these models cannot capture aspects of human-specific development that may be important for understanding human brain function. hBO models of the human brain offer a step towards achieving the ambitious goal of modeling the complex development of the human brain in a dish. Combining the knowledge gained from patient genetics with hBO technology offers a platform for testing how altering the expression of disease-associated genes impacts cellular signaling, development, and neuronal activity in the context of human neural development. Modeling somatic mutations in hBOs will provide a new system for testing how these mutations cause brain malformations, how different mutations and their time of onset can change the cellular composition of the developing brain, and how cells harboring somatic mutations contribute to the altered function of the surrounding brain circuitry.

## Author Contributions

HB conceived the idea for the review. BD wrote the initial draft. BD and HB edited the manuscript and designed the figures. All authors contributed to the article and approved the submitted version.

## Conflict of Interest

The authors declare that the research was conducted in the absence of any commercial or financial relationships that could be construed as a potential conflict of interest.

## Publisher’s Note

All claims expressed in this article are solely those of the authors and do not necessarily represent those of their affiliated organizations, or those of the publisher, the editors and the reviewers. Any product that may be evaluated in this article, or claim that may be made by its manufacturer, is not guaranteed or endorsed by the publisher.
